# Effects of tolvaptan on urine output in hospitalized heart failure patients with hypoalbuminemia or proteinuria

**DOI:** 10.1007/s00380-017-1066-4

**Published:** 2017-10-23

**Authors:** Koji Takagi, Naoki Sato, Shiro Ishihara, Michiko Sone, Hideo Tokuyama, Kenji Nakama, Toshiya Omote, Arifumi Kikuchi, Masahiro Ishikawa, Kenichi Amitani, Naoto Takahashi, Yuji Maruyama, Hajime Imura, Wataru Shimizu

**Affiliations:** 1 Cardiology and Intensive Care Unit, Nippon Medical School Musashi-Kosugi Hospital, Musashi-Kosugi, 1-396 Kosugi-cho, Nakahara-ku, Kawasaki, Kanagawa 211-8533 Japan; 2 Department of Cardiovascular Surgery, Nippon Medical School Musashi-Kosugi Hospital, Musashi-Kosugi, Kawasaki, Japan; 3 Department of Cardiovascular Medicine, Nippon Medical School, Tokyo, Japan

**Keywords:** Hypoalbuminemia, Proteinuria, Diuretic resistance, Tolvaptan

## Abstract

Hypoalbuminemia is an independent prognostic factor in hospitalization for heart failure (HHF). Hypoalbuminemia or proteinuria is related to resistance to loop diuretics. Tolvaptan is an oral non-peptide, competitive antagonist of vasopressin receptor-2. It has been used for the treatment of volume overload in HHF patients in several Asian countries. Several studies have demonstrated marked improvement in congestion in HHF patients. However, whether tolvaptan is useful for HHF patients with hypoalbuminemia or proteinuria (both of which are related to resistance to loop diuretics) has not been clarified. We examined the diuretic response to tolvaptan in HHF patients with hypoalbuminemia or proteinuria. We defined hypoalbuminemia as a serum level of albumin < 2.6 g/dl. Fifty-one HHF patients who received additional tolvaptan upon therapies with loop diuretics were divided into the hypoalbuminemia group (*n* = 24) or control group (*n* = 27). The changes in urine output per day were not different between the two groups [610 (range 100–1032); 742 (505–1247) ml, *P* = 0.313]. There was no difference in diuretic responses between patients with and without proteinuria. The serum level of albumin did not correlate with changes in urine output per day after tolvaptan treatment (*P* = 0.276, *r* = 0.156). Thus, additional administration of tolvaptan elicited a good diuretic response in HHF patients with hypoalbuminemia or proteinuria. These data suggest that tolvaptan might be beneficial for such HHF patients.

## Introduction

Hypoalbuminemia can be caused by malnutrition, inflammation, hemodilution, nephrotic syndrome, or cachexia. Hypoalbuminemia is an independent prognostic factor in hospitalization for heart failure (HHF) [1–3]. Hypoalbuminemia is related to the resistance of loop diuretics, e.g., urinalysis [4], as a result of decreased delivery of drugs to the kidneys, although a bolus injection of furosemide with albumin [4] or continuous infusion of this combination [5] has been reported to improve diuresis in patients with hypoalbuminemia. Proteinuria is also one of the mechanisms of the diuretic resistance [6] and poor outcome of HHF [7, 8]. Thus, it has been well known that hypoalbuminemia or proteinuria is an important factor of diuretic resistance [9].

Tolvaptan is an oral non-peptide, selective antagonist of vasopressin receptor-2. Tolvaptan is used for the treatment of the following: hyponatremia in the United States; syndrome of inappropriate secretion of antidiuretic hormone in the European Union; and volume overload in patients with heart failure in several Asian countries. Several clinical trials and studies have demonstrated marked improvement of congestion in HHF patients [10, 11]. According to Starling’s law, a low plasma oncotic pressure due to hypoalbuminemia induces a fluid shift from the intravascular space to the interstitial space, and there is clinical evidence that hypoalbuminemia facilitates the onset of cardiogenic pulmonary edema [12]. Tolvaptan produces a free-water diuresis and could increase osmotic pressure in the acute setting. Therefore, it is thought that the net effect of tolvaptan could produce a sustained movement of fluid from the extravascular space to the vascular space [13]. Those observations suggest that, with appropriate urinalyses, tolvaptan may help to improve congestion. In liver cirrhosis, tolvaptan has been reported to improve hepatic edema independent of the serum level of albumin [14]. However, the efficacy of tolvaptan has not been examined thoroughly in HHF patients with relatively moderate-to-severe hypoalbuminemia or proteinuria. The aim of the present study was to examine the diuretic effect of tolvaptan in such HHF patients.

## Materials and methods

The study protocol was approved by the ethics committee of Nippon Medical School Musashi-Kosugi Hospital (Kanagawa, Japan). The present study had a retrospective design and was undertaken to examine the urinary effects of tolvaptan in HHF patients with and without hypoalbuminemia or proteinuria. All patients admitted to the Nippon Medical School Musashi-Kosugi Hospital with HHF and who received tolvaptan from June 2011 to April 2013 were enrolled. Heart failure was diagnosed by medical teams using the Framingham criteria [15]. The patients with diuretic resistance with natriuretic diuretics were enrolled. The enrolled patients included HHF due to ischemic causes and three patients with acute coronary syndrome. However, administrations with tolvaptan were not performed at acute phase. Choices of diuretics and dose adjustments were made based upon the clinical judgment of these medical teams. Diuretic dose was corrected to be an equivalent dose of furosemide [16–18]. Patient characteristics as well as physical and laboratory data were examined retrospectively using medical records. The goal of this study was to ascertain if the changes in 24-h urine output just before and after tolvaptan administration in patients with and without hypoalbuminemia or proteinuria are different. “Hypoalbuminemia” was defined as a serum level of albumin < 2.6 g/dl, which was defined to be a “medium” level in the present study. The classic definition for hypoalbuminemia is less than 3.0 g/day, but the definitions of the previous studies vary from < 2.5 to < 3.5 g/dl [14, 19]. Based on these references, hypoalbuminemia in the present study was simply defined as a serum level of albumin < 2.6 g/dl, which was defined to be a “medium” level. The estimated glomerular filtration rate (eGFR) was calculated using the Modification of Diet in Renal Disease equation [20]. Proteinuria was measured by a point-of-care test tape (Uriflet S; Arkray, Kyoto, Japan). The corresponding author had full access to all study data, and takes responsibility for the integrity of data and the accuracy of data analyses.

### Statistical analyses

Analytical data are the mean ± standard deviation (SD) or median with 25th and 75th percentiles for continuous variables, whereas categorical variables are presented as numbers or percentages. Continuous variables were compared using Student’s *t* test or the Wilcoxon rank-sum test for two-group comparisons. Categorical variables were compared using Fisher’s exact test. *P* < 0.05 was considered significant. To determine predictors of urine output in response to tolvaptan, multivariate analysis was performed in both separated variable sets including serum albumin or proteinuria, because these two variables were not independent. Clinical variables included age, sex, eGFR, serum sodium, left ventricular ejection fraction, and serum albumin or urine protein. Statistical analyses were done using R v3.1.1 (R Development Core Team, Vienna, Austria).

## Results

**Fig. 1:**
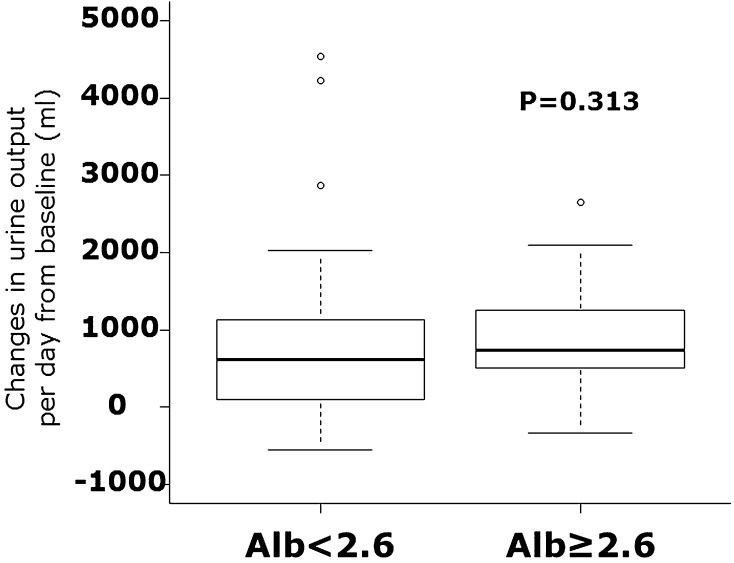
Comparison of changes in urine output per day from baseline (before tolvaptan administration) between hypoalbuminemia (albumin level < 2.6 g/dl, left) and control (≥ 2.6 g/dl, right) is shown. There were no differences between two groups. *Alb* albumin

**Fig. 2:**
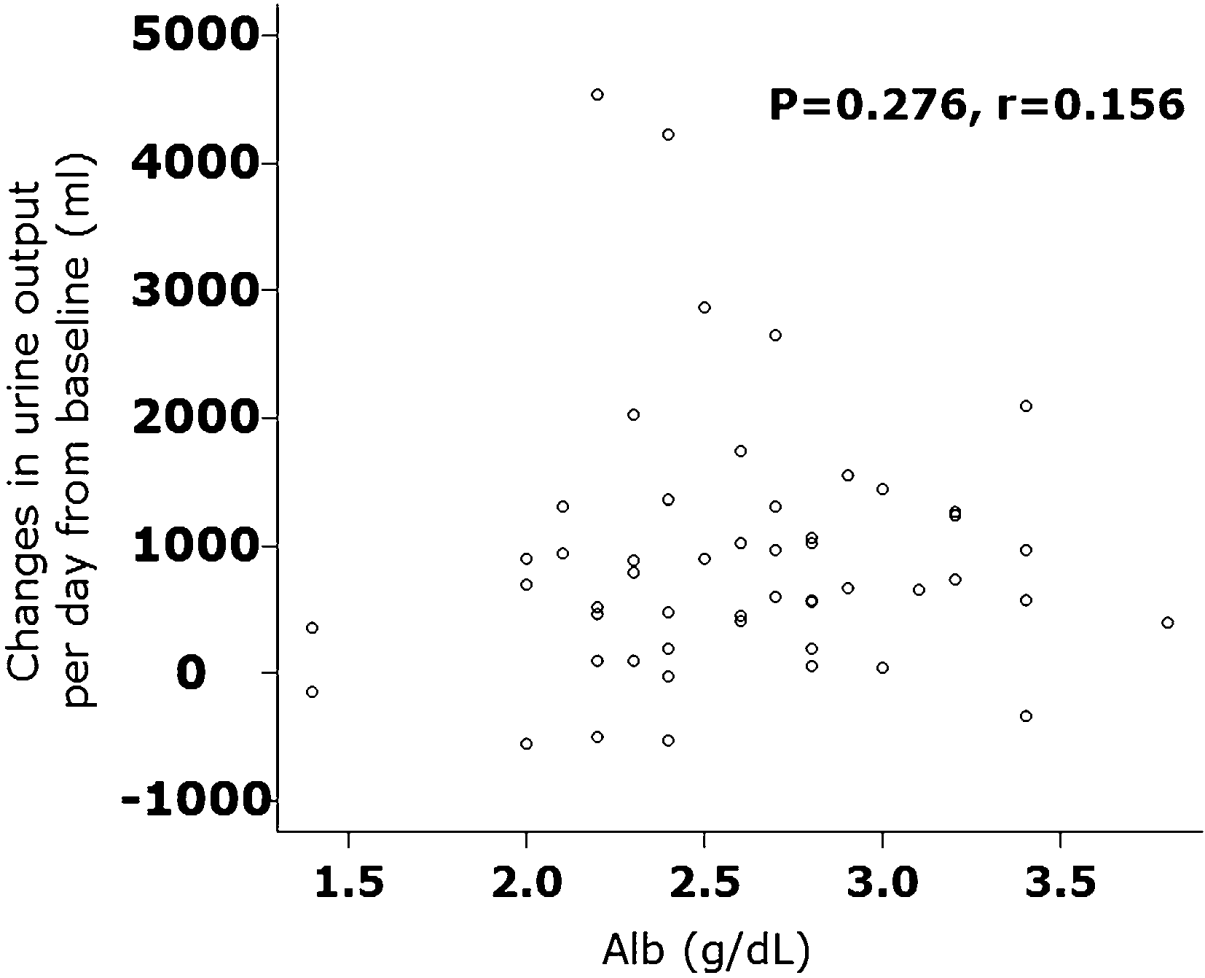
Relationship between changes in urine output per day from baseline and serum albumin (Alb) is shown. There was statistically no relationship between these parameters, suggesting that tolvaptan can excrete urine irrespective of serum albumin levels

Fifty-one patients were included in the present study, of which 24 patients were defined as having hypoalbuminemia. Baseline characteristics are shown in Table 1. The enrolled patients included preserved and reduced left ventricular ejection fraction (≥ 50%; *n* = 27, < 50%; *n* = 24). No significant differences in baseline data were observed except for levels of albumin and C-reactive protein, proportion of patients with dyslipidemia, and use of spironolactone. For patients with hypoalbuminemia, the mean level of albumin was 2.2 ± 0.3 (2.1–2.4) g/dl. For the control group, the mean level of albumin was 3.0 ± 0.3 (2.7–3.2) g/dl. Urine output before and after tolvaptan administration was increased significantly in each group. The effect of tolvaptan on changes in urine output for patients with and without hypoalbuminemia is shown in Fig. 1. The changes in urine output per day and systolic blood pressure were not different between the two groups. The serum level of albumin did not correlate with changes in urine output per day after tolvaptan treatment (*P* = 0.276, *r* = 0.156) (Fig. 2).

**Table 1:** Demographic and clinical characteristics

	Albumin < 2.6 g/dl (*n* = 24)	Albumin ≥ 2.6 g/dl (*n* = 27)	*P* value
Age, years	77.7 ± 13.9	76.3 ± 13.1	0.711
Male	10 (41.7%)	11 (40.7%)	1.000
Comorbidity condition			
Hypertension	17 (70.8%)	18 (66.7%)	0.772
Diabetes mellitus	9 (37.5%)	10 (37.0%)	1.000
Dyslipidemia	4 (16.7%)	12 (44.4%)	0.040
Atrial fibrillation	8 (33.3%)	14 (51.9%)	0.259
Etiology			
Ischemic	7 (29.2%)	8 (29.6%)	1.000
Dilated cardiomyopathy	3 (12.5%)	3 (11.1%)	1.000
Hypertensive heart	1 (4.2%)	3 (11.1%)	0.612
Valvular disease	6 (25.0%)	8 (29.6%)	0.762
Arrhythmia	2 (8.3%)	1 (3.7%)	0.595
Others	5 (20.8%)	4 (14.8%)	0.718
LVEF (%)	43.4 ± 17.9	50.9 ± 19.6	0.166
Laboratory values			
Hemoglobin (g/dl)	9.8 ± 2.0	10.5 ± 2.1	0.175
Albumin (g/dl)	2.2 ± 0.3	3.0 ± 0.3	< 0.001
BUN (mg/dl)	35.1 ± 24.9	31.5 ± 17.9	0.556
Creatinine (mg/dl)	1.61 ± 1.47	1.33 ± 1.39	0.483
eGFR (ml/min/1.73 mm^2^)	54.4 ± 38.7	54.8 ± 32.9	0.967
Sodium (mEq/l)	133 ± 8.1	135.3 ± 5.5	0.179
Potassium (mEq/l)	4.2 ± 0.7	4.4 ± 0.6	0.469
Chloride (mEq/l)	97.5 ± 8.5	97.1 ± 5.7	0.830
CRP (mg/dl)	6.96 ± 5.39	3.41 ± 3.16	0.005
NT-proBNP (pg/ml), median (25–75 percentiles)	7403 (2982–9000)	5303 (1417–8875)	0.097
Urine protein (dipstick test)			
Negative	12 (23.5%)	18 (58.8%)	0.516
1 +	7 (13.7%)	6 (11.8%)	
2 +	4 (7.8%)	3 (5.9%)	
3 to 4 +	1 (2.0%)	0 (0.0%)	
Vital signs			
Heart rate, beats/min	84.4 ± 18.4	81.0 ± 14.0	0.451
Systolic BP, mmHg	121 ± 17.4	113.1 ± 17.4	0.116
Diastolic BP, mmHg	58.9 ± 11.9	58.5 ± 10.0	0.897
Dose of diuretics			
Tolvaptan (mg/day)	6.4 ± 2.6	5.3 ± 2.6	0.128
Furosemide (mg/day)	73.3 ± 47.7	74.8 ± 42.8	0.907
Furosemide iv (mg/day)	47.8 ± 19.8	40.9 ± 26.6	0.446
Other diuretics			
Furosemide	22 (91.7%)	24 (88.9%)	1.000
Azosemide	0 (0%)	4 (14.8%)	0.113
Torasemide	0 (0%)	5 (18.5%)	0.052
Trichlormethiazide	0 (0%)	1 (3.7%)	1.000
Spironolactone	7 (29.2%)	16 (59.3%)	0.048

Data are presented as mean ± SD or *n* (%)

*LVEF* left ventricular ejection fraction, *BUN* blood urea nitrogen, *eGFR* estimated glomerular filtration rate, *CRP* C-reactive protein, *NT-proBNP* N-terminal pro-B-type natriuretic peptide, *BP* blood pressure, *iv* intravenous

**Fig. 3:**
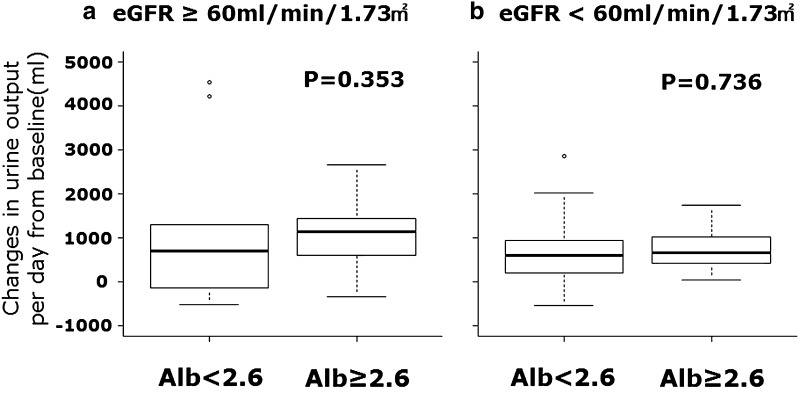
Comparisons of changes in urine output per day from baseline between hypoalbuminemia and control groups in preserved renal function (estimated glomerular filtration ratio (eGFR) ≥ 60 ml/min/1.73 m^2^, **a**) and impaired renal function (eGFR < 60 ml/min/1.73 m^2^, **b**) are shown. The changes in urine output per day from baseline between two groups in the patients with and without impaired renal function, suggesting that tolvaptan can be effective in terms of urine excretion irrespective of renal function

**Fig. 4, 5:**
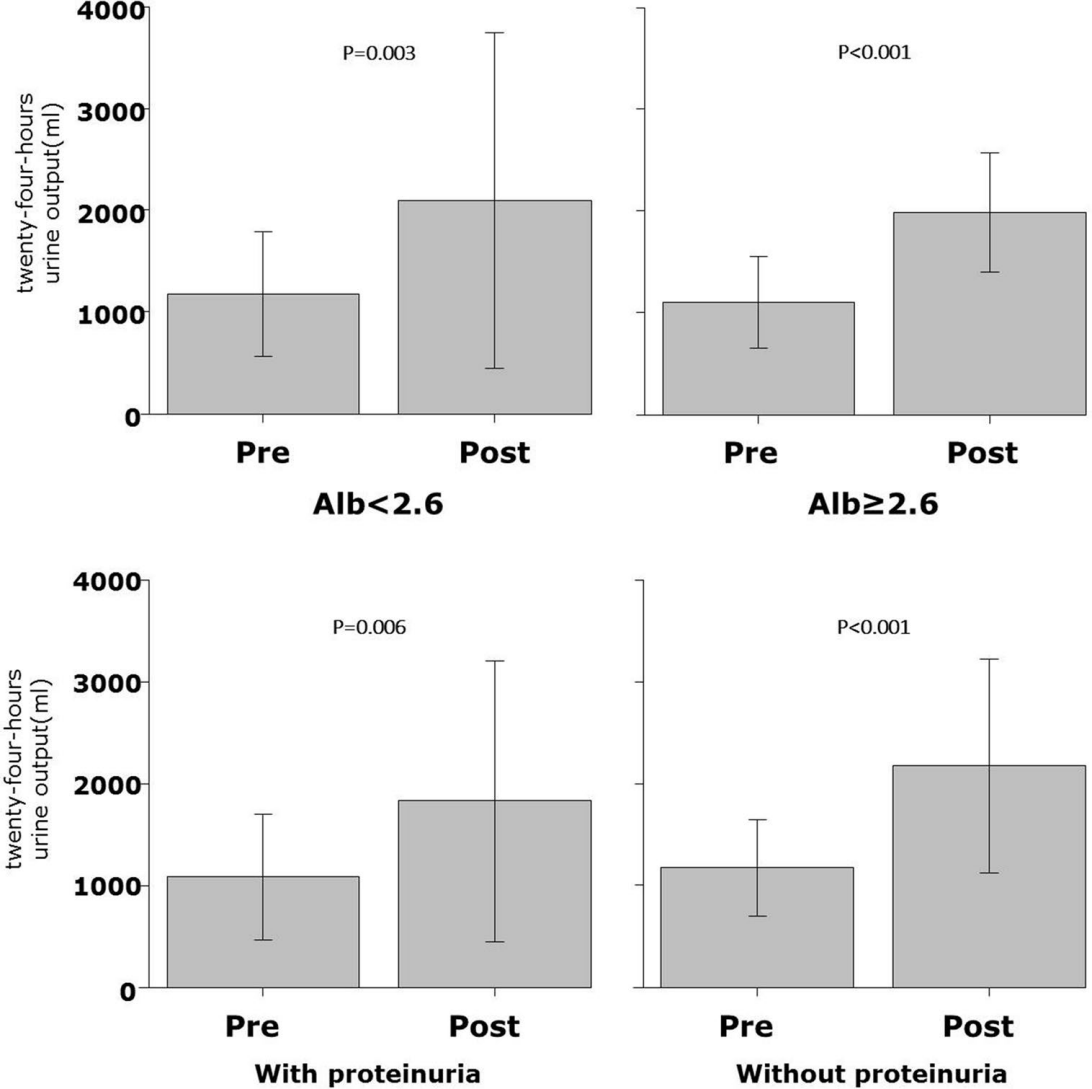
Stratified analysis of 24-h urine output. Data are expressed as mean ± standard deviation. Comparisons between before and after tolvaptan administration in with and without hypoalbuminemia or with and without proteinuria and were performed using the paired *t* test

**Fig. 6:**
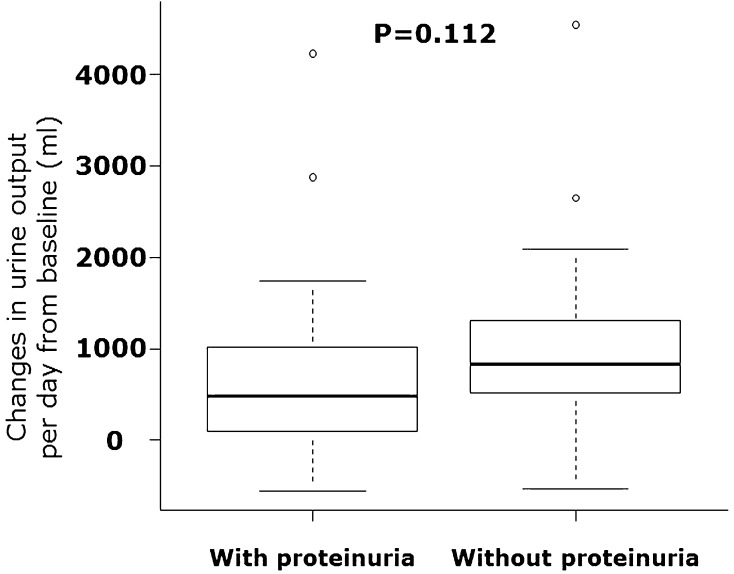
Comparison of changes in urine output per day from baseline (before tolvaptan administration) between with and without proteinuria is shown. There were no differences between two groups

The changes in urine output per day were not different before and after tolvaptan administration in normal renal function [defined as eGFR ≥ 60 ml/min/1.73 m^2^ (Fig. 3a, + 701(range – 88 to 1209) ml in hypoalbuminemia vs. + 1128 (693–1405) ml in control, *P* = 0.353] and impaired renal function (< 60 ml/min/1.73 m^2^) [Fig. 3b, + 588 (range 237–929) ml in hypoalbuminemia vs. + 651 (414–1024) ml in control, *P* = 0.736)]. Furthermore, in patients with proteinuria (*n* = 21), the changes in urine output per day were not different between the two groups [+ 476 (range 100–1020) ml with proteinuria vs. + 834 (534–1309) ml without proteinuria, *P* = 0.112]. Urine output before and after tolvaptan administration was increased significantly in both groups with hypoalbuminemia on the left side of Fig. 4 (1180 ± 611 ml before tolvaptan vs. 2092 ± 1650 ml after tolvaptan, *P* = 0.003) and without hypoalbuminemia on the right side of Fig. 4 (1102 ± 457 ml before tolvaptan vs. 1988 ± 585 ml after tolvaptan, *P* < 0.001). Figure 5 shows the results of changes in urine output before and after tolvaptan in the patients with and without proteinuria (Fig. 5, 1086 ± 612 ml before tolvaptan vs. 1836 ± 1381 ml after tolvaptan, *P* = 0.006, 1175 ± 474 ml before tolvaptan vs. 2178 ± 1051 ml after tolvaptan, *P* < 0.001, respectively). The effect of tolvaptan on changes in urine output for patients with and without proteinuria is shown in Fig. 6, which demonstrated no significant difference in urine output changes between both groups (*P* = 0.112), although changes in urine output in response to tolvaptan in proteinuria tended numerically to be less compared with those in the patients without proteinuria. Furthermore, multivariate analysis showed that serum albumin and urine protein were not significantly predictors for urine out in response to tolvaptan, although eGFR was the only independent predictor in the model with serum albumin [*R* ^2^ = 0.09, 9.23, 95% confidence intervals (CI) (0.148–18.311), *P* = 0.047] and with urine protein [*R* ^2^ = 0.09, *β* 9.55, 95% CI (− 0.009 to 19.105), *P* = 0.05].

## Discussion

Hypoalbuminemia or proteinuria is a common finding and a major cause of diuretic resistance (especially for loop diuretics) in HHF patients. The present study demonstrated no differences in the urinary effects of the aquaretic diuretic tolvaptan regardless of serum levels of albumin or proteinuria.

Volume overload is an essential target for HHF treatment [21]. Increased left ventricular filling pressure can lead to volume overload owing mainly to an increase in hydrostatic pressure and low oncotic pressure in plasma based on Starling’s law. A low oncotic pressure in plasma associated with hypoalbuminemia can induce congestion in HHF patients [12] and resistance to loop diuretics [22]. Owing to these physiologic conditions in HHF patients, an appropriate approach should be considered for diuretic management of HHF patients with hypoalbuminemia.

Furosemide is used most commonly for the management of HF. Furosemide binds strongly to proteins (mainly to albumin) and is secreted into the lumen of the proximal tubule by organic acid transporters. Hypoalbuminemia means that fewer albumin molecules can bind to furosemide and that a smaller amount of furosemide is delivered to the proximal tubule, which induces diuretic resistance. Therefore, a high dose of furosemide is needed to control volume overload in HHF patients. This high dose causes the pathophysiologic condition of HF to deteriorate via neurohumoral activation and increases the risk of death [18]. Combination therapy with furosemide and albumin might be possible for patients with hypoalbuminemia, but evidence to clarify the clinical significance of such combination therapy is lacking [4, 23], even in patients with liver cirrhosis [24].

Tolvaptan can improve volume overload, symptoms, and signs, and does not impair renal function in HHF patients [10, 11]. A phase-III, multicenter, randomized, double-blind, placebo-controlled trial demonstrated that tolvaptan reduced body weight significantly in patients with hepatic edema and low serum level of albumin (< 2.5 g/dl), suggesting that tolvaptan could improve hepatic edema independent of the serum level of albumin [14]. Several studies have demonstrated that tolvaptan can increase urine output in patients with heart failure and hypoalbuminemia [25] and in patents with liver cirrhosis and hypoalbuminemia [26]. The present study also showed the importance of tolvaptan in HHF patients with moderate-to-severe hypoalbuminemia. In addition, we demonstrated that tolvaptan is effective in HHF patients with proteinuria, though a recent study revealed albuminuria to be a factor of renal resistance to loop diuretics [27]. Taken together, tolvaptan could be useful for the management of hypoalbuminemia or proteinuria.

The mechanism by which tolvaptan is efficacious against hypoalbuminemia is not known. A pharmacokinetic study in vitro revealed tolvaptan to bind strongly to plasma proteins in humans [28], so (i) there may be some differences in protein-binding status in vivo and in vitro; (ii) protein-free tolvaptan might bind strongly to vasopressin receptor-2 and increase urine output. Further investigations are needed to answer this question fully. It has not been investigated whether or not tolvaptan is effective in the patients with proteinuria. This study was the first study to investigate it. Several case reports have demonstrated diuretic efficacy of tolvaptan in nephrotic syndrome [29, 30]. In the patients with autosomal dominant polycystic kidney disease, tolvaptan improved renal function [31]. In this trial, the subjects included the patients with proteinuria at about one-fourth. These data might suggest the beneficial efficacy of tolvaptan in the patients with proteinuria. Two mechanisms of this efficacy could be considered. First, natriuretic effects of tolvaptan have been demonstrated via the decreases in sodium reabsorption against the effect of vasopressin with the activation of epithelial Na-channels in the distal nephron [32, 33]. Second, tolvaptan restored reportedly podocyte injury in nephrotic rats [34], which had intraglomerular hypertension by podocyte injury via activation of vasopressin. Thus, tolvaptan might induce effective diuresis and also protect renal injury.

The present study had limitations. First, the present study conducted at the single center with a small cohort and indication of tolvaptan by the limited physicians; therefore, the selection of the patients might have some bias; however, we enrolled consecutive cases and managed by the medical teams consisting of different physicians to reduce selection bias as much as possible. Second, this was a retrospective study, but there were no exclusion criteria and we included HHF patients regardless of left ventricular ejection fraction with relatively more severe hypoalbuminemia compared with previous studies. Furthermore, the present study examined in the patients with proteinuria. Third, the present study demonstrated that no relationship between serum albumin levels and changes in urine output after tolvaptan administration without the control group, because it has been well recognized that the diuretic response to furosemide is impaired in the patient with hypoalbuminemia or proteinuria [9]. This limitation should be clarified by a future prospective study. Fourth, urinary protein was estimated using a point-of-care test in the present study. Although the gold standard for measurement of protein excretion is a 24 h-urine collection, several studies have already demonstrated that proteinuria of 1 + or greater by a point-of-care test is reliable to detect significant proteinuria in spot urine samples with a high sensitivity and specificity [35]. Furthermore, a point-of-care test is easily used in clinical setting. Therefore, a point-of-care test for the detection of proteinuria in the present study was chosen in terms of availability for clinical setting. Fifth, multivariate analysis showed that both serum albumin and urine protein were not statistically independent predictors for urine output in response to tolvaptan, although eGFR was the independent predictor. However, the *R* ^2^ was low in both analysis; therefore, the results of the present study should be confirmed by further studies. Sixth, in the present study, serum sodium levels were measured by indirect potentiometry, which is influenced by levels of protein and albumin. Therefore, the data and the results of analysis could be influenced in the present study. On the other hand, serum sodium levels are also influenced by vasopressin levels. Therefore, the diuretic response to tolvaptan could be influenced by serum sodium levels. However, the previous studies have demonstrated that there was no correlation between diuretic responses to tolvaptan and serum sodium levels in HF [32] as well as liver cirrhosis [36]. In the present study, there was no correlation between serum sodium levels and changes in urine output in response to tolvaptan (not shown in the results). From these considerations, it could be difficult to correct serum sodium levels using serum albumin levels in HHF patients. Seventh, the results should be confirmed by controlled studies in the patients with hypoalbuminemia and/or proteinuria treated by furosemide with and without additional tolvaptan. Finally, tolvaptan was administrated in cases of diuretic resistance with furosemide in the present study. If the timing of administration of tolvaptan was changed to an earlier period, a different response to tolvaptan might have been seen. However, we think that the results from the present study would be representative of real-world clinical settings. This belief can be confirmed by large-scale randomized studies compared between the patients, who have hypoalbuminemia and/or proteinuria, treated by furosemide with and without tolvaptan.

In conclusion, the present study showed the efficacy of tolvaptan in terms of urine output in HHF patients with hypoalbuminemia and proteinuria. Combination of tolvaptan with furosemide could be a useful therapeutic strategy for HHF patients with hypoalbuminemia or proteinuria.
